# Giant Kerr nonlinearity of terahertz waves mediated by stimulated phonon polaritons in a microcavity chip

**DOI:** 10.1038/s41377-024-01509-y

**Published:** 2024-08-23

**Authors:** Yibo Huang, Yao Lu, Wei Li, Xitan Xu, Xinda Jiang, Ruobin Ma, Lu Chen, Ningjuan Ruan, Qiang Wu, Jingjun Xu

**Affiliations:** 1 The Key Laboratory of Weak-Light Nonlinear Photonics, Ministry of Education, TEDA Applied Physics Institute and School of Physics, Nankai University, Tianjin, 300071 China; 2 Shenzhen Research Institute of Nankai University, Shenzhen, 518083 Guangdong China; 3 Beijing Institute of Space Mechanics & Electricity, China Academy of Space Technology, 100094 Beijing, China

**Keywords:** Terahertz optics, Nonlinear optics, Polaritons, Micro-optics, Ultrafast photonics

## Abstract

Optical Kerr effect, in which input light intensity linearly alters the refractive index, has enabled the generation of optical solitons, supercontinuum spectra, and frequency combs, playing vital roles in the on-chip devices, fiber communications, and quantum manipulations. Especially, terahertz Kerr effect, featuring fascinating prospects in future high-rate computing, artificial intelligence, and cloud-based technologies, encounters a great challenge due to the rather low power density and feeble Kerr response. Here, we demonstrate a giant terahertz frequency Kerr nonlinearity mediated by stimulated phonon polaritons. Under the influences of the giant Kerr nonlinearity, the power-dependent refractive index change would result in a frequency shift in the microcavity, which was experimentally demonstrated via the measurement of the resonant mode of a chip-scale lithium niobate Fabry-Pérot microcavity. Attributed to the existence of stimulated phonon polaritons, the nonlinear coefficient extracted from the frequency shifts is orders of magnitude larger than that of visible and infrared light, which is also theoretically demonstrated by nonlinear Huang equations. This work opens an avenue for many rich and fruitful terahertz Kerr effect based physical, chemical, and biological systems that have terahertz fingerprints.

## Abstract (web summary)

We demonstrate a giant terahertz frequency Kerr nonlinearity mediated by stimulated phonon polaritons, which provides a new avenue for the future terahertz technologies.

## Graphical abstract

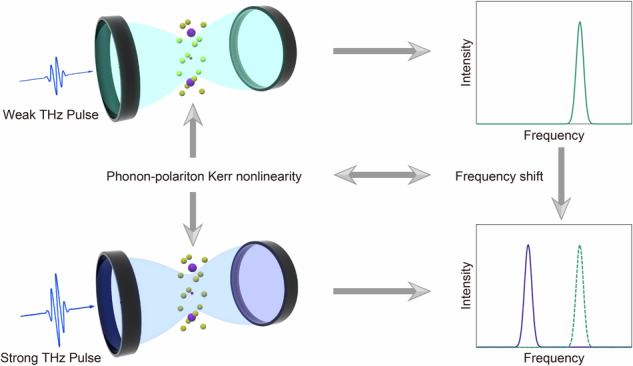

## Introduction

Terahertz (THz) wave, which bridges the gap between microwave and infrared light, is a burgeoning and promising field, researches on THz technologies^1–4^ such as radiation^5^, detection^6,7^, imaging^8,9^, and communications^10^, have yielded significant successes in recent years. With the advancement of THz technologies, studies on THz nonlinear optics have emerged and achieved considerable breakthroughs, including high-harmonic generation^11^, induced ferroelectricity^12^, electron controlling^13,14^, and phonon modulation^15,16^. In the past decades, the Kerr effect has given birth to various nonlinear phenomena, such as optical solitons^17,18^, self-phase modulation^19^ and cross-phase modulation^20^. Furthermore, it has facilitated the development of various technologies, including supercontinuum spectra^21^, frequency combs^22,23^, all-optical switching^24^, optical isolator^25^, and all-optical modulator^26^. The urgent desire for THz Kerr effect (TKE) not only lies in the investigations and achievements of the above physics and technologies at the THz-frequency band, but it can also provide opportunities to realize high-speed wireless communications, high-rate computing, THz nonlinear photonic chips, artificial intelligence, and many cloud-based technologies.

However, the studies on TKE cannot support such a fascinating blueprint yet. In 2019, it has been demonstrated that water exhibits a high nonlinear refractive index coefficient at THz frequency owing to the contribution of molecular vibrations^27^. Although experiments have suggested that thermal effects or acceleration of carriers can induce an increase in the TKE nonlinearity in silicon at high THz intensities^28^, it is still feeble and impedes practical THz applications. On the other hand, THz pulses can also induce or enhance the Kerr effect of visible and infrared light in nonpolar liquids^29^, air^30^, and glasses^31^, but the nonlinearity remains weak and is still distant from practical applications. The advancement of TKE necessitates new mechanisms and new methods.

For visible and near-infrared light, Kerr nonlinearity is considered to mainly originate from the electronic nonlinearity, whereas for microwaves or THz waves, the ionic contribution cannot be ignored. Theoretical predictions suggest that the contribution of ionic nonlinearity is larger than that of electronic one for microwave and THz frequencies^32,33^. Importantly, the contribution of the stimulated phonon polaritons (SPhPs), which would be excited if THz waves enter and strongly couple with polar optical phonons in ionic crystals^34^, would dominate the THz nonlinear effects in many cases^33,35^. This is caused by a novel light-matter interaction mechanism mediated by SPhPs^35^, in which SPhPs show remarkable delocalization and coherence, and thereby the THz nonlinear effects are greatly enhanced^33^.

In this work, we demonstrated a giant TKE in a chip-scale lithium niobate (LN) Fabry-Pérot microcavity mediated by SPhPs. Under the influence of the giant TKE, the power-dependent refractive index changes resulted in frequency shifts of the resonant modes in a single-mode microcavity, which were experimentally measured and analyzed. The experimentally SPhP-enhanced third-order nonlinear optical susceptibility is demonstrated several magnitudes larger than that in visible or infrared frequency, consistent well with the theoretical predictions from the nonlinear Huang equations^33,35^. Besides, the frequency shifts caused by the hybrid modulation (TKE and cross-modulation) in the multi-mode microcavity also align well with the theoretical predictions. Our results provide a new avenue for technologies and applications based on TKE.

## Results

**Fig. 1:**
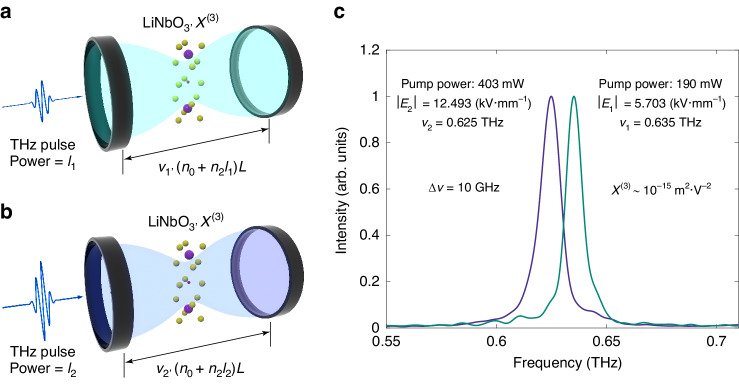
Terahertz Kerr effect induced frequency shift. **a**, **b** THz waves strongly couple with phonons in ionic crystals, taking lithium niobate for an example, resulting in a remarkable increase in the Kerr nonlinearity, which modulates the refractive index, leading to a frequency shift. **c** Spectrum of a single-mode microcavity at pump powers of 190 mW and 403 mW. The initial peak amplitudes are 5.703 kV mm^−1^ and 12.493 kV mm^−1^, respectively, and the corresponding frequencies are 0.635 and 0.625 THz, and the third-order nonlinear optical susceptibility can be calculated from the frequency shift

To better clarify the TKE in a microcavity, we consider an ideal one-dimensional Fabry-Pérot cavity filled with a Kerr nonlinear medium (LN typically), as shown schematically in Fig. 1a, b. The resonant wavelength $\lambda$ of the cavity (with a length of $L$) satisfies $M\lambda =2{nL}$, where $n$ denotes the effective refractive index of the medium within the cavity, with $M$ being a positive integer. For a Kerr medium, the refractive index $n$ should consist of a linear component, ${n}_{0}$, and a nonlinear component, ${n}_{2}I$, where $I$ represents the input THz power. Hence the resonant frequency $\nu$ is determined by:

1 $$\nu \left(I\right)=\frac{{Mc}}{2\left({n}_{0}+{n}_{2}I\right)L}$$

where $c$ is the speed of light in vacuum, ${n}_{2}$ is the Kerr coefficient. This result indicates that the resonant frequency of a Kerr microcavity would change with the input THz power. In Fig. 1c, an experimental frequency-shift result of 10 GHz (about 1.5%) is displayed.

**Fig. 2:**
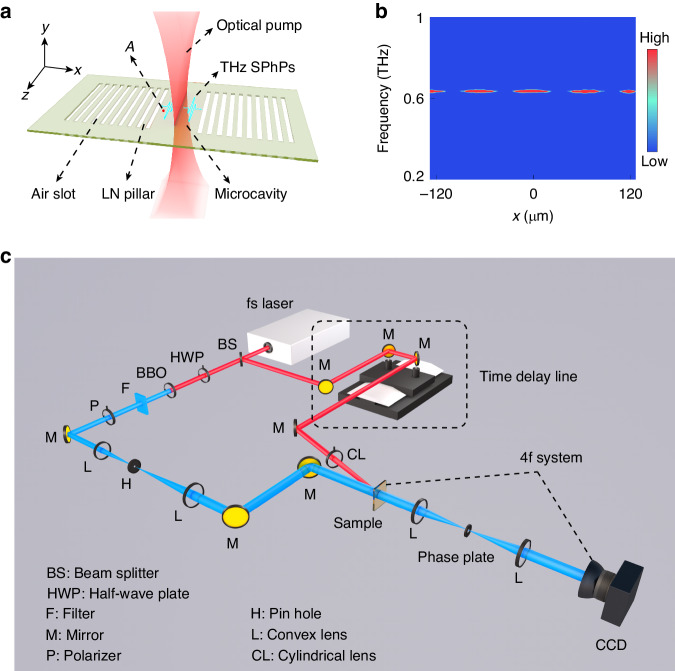
Schematic diagram of a microcavity and the experimental setup. **a** Schematic diagram of a Fabry-Pérot microcavity, the cavity is sandwiched between two distributed Bragg reflectors, each Bragg reflector is made up of air slots and LN pillars arranged periodically. A beam of laser is focused into the cavity to generate THz waves. The time traces of the THz *E*-field in the microcavity are recorded, at point *A*, 60.75 μm away from THz excitation line. **b** Spectral information obtained by applying Fourier transformation to the electric fields in a single-mode microcavity. **c** Schematic of the experimental pump-probe time-resolved imaging system

In the experiment, a Fabry-Pérot microcavity is fabricated on a 50 μm-thick *x*-cut MgO:LiNbO_3_ (LN) slab waveguide using the femtosecond laser direct writing technique (see Supplementary Note 1). The microcavity is sandwiched between two distributed Bragg reflectors (DBRs) that serve as mirrors^36^, as schematically shown in Fig. 2a. Each DBR is constructed through the alternation of air slots and LN pillars, with energy bands being tailored by adjusting the periods and dimensions of these features. THz waves in the microcavity are generated by femtosecond laser pulses via nonlinear effects, ranging from 0.2–1.2 THz in frequency. The specific modes in a microcavity can be manipulated by designing the DBRs, selecting the excitation position, and adjusting the microcavity length. In order to clearly investigate the TKE in the microcavity, a single-mode microcavity was designed with a length of 245 μm, in which the resonant mode is located at 0.63 THz. The air slots in the DBRs have a width of 50 μm, and the distance between each adjacent slot is 100 μm. By focusing the pump pulses in the center of the microcavity, the single-mode microcavity is excited. Figure 2b shows the spectral information in the microcavity, indicating the excitation of the single mode, and the resonant frequency of the microcavity, $\nu$, satisfies:

2 $$M\frac{c}{\nu }=2\left({n}_{0}+{n}_{2}I\right)L$$

Here, ${n}_{0}$ is the linear part of effective refractive index of the LN microcavity chip^37^, and ${n}_{2}I$ is the nonlinear part.

In the experiment, THz waves were generated and detected in the microcavity utilizing the pump-probe technique^7,38^, as displayed in Fig. 2c. Femtosecond laser pulses (800 nm central wavelength, 120 fs duration, 1 kHz repetition rate) are split into two branches using a beam splitter, in which the pump beam (190–403 μJ) is cylindrically focused into the microcavity to generate THz waves (see “Materials and methods”), while the probe beam (50 μJ) is frequency doubled and delayed for recording the generated THz waves. When the probe beam passes through the sample, the generated THz waves in the LN microcavity induce a refractive index change via the electro-optic effect (see “Materials and methods”), therefore the probe beam gets a phase shift proportional to the refractive index distribution. Then the probe beam passes through a phase-contrast system to convert phase distribution to intensity distribution, and is finally collected by a CCD camera. The time delay between the pump and probe pulses is adjusted by moving the mechanical delay line, then the full spatiotemporal evolution of THz waves, that is, the electric field oscillation in the microcavity, is obtained from the image sequence. Spectral information in the microcavity can be obtained by applying Fourier transformation to the electric fields.

**Fig. 3:**
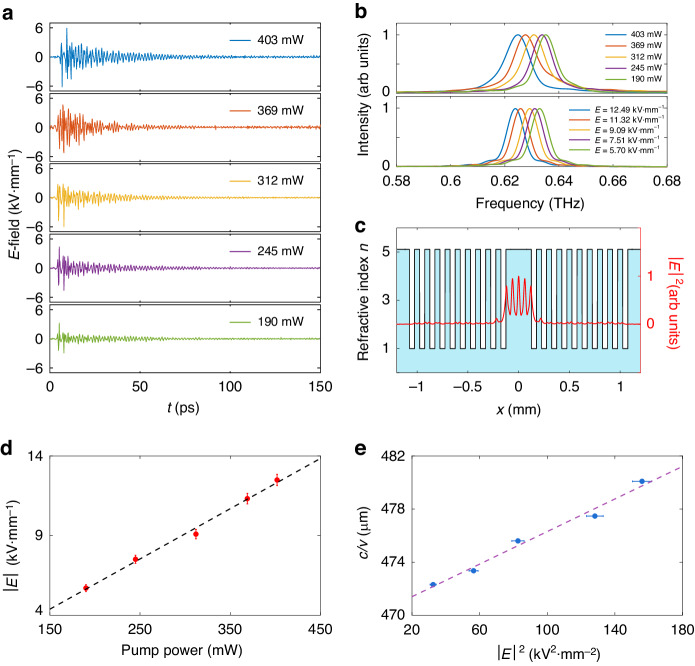
Frequency shift in a single-mode microcavity. **a** Time traces of the THz *E*-field at point A in the microcavity with varied pump powers. **b** Top panel: spectra of a LN microcavity at different pump power; bottom panel: simulation results of frequency shifts. **c** Cavity structures and the calculated field intensity distribution of the resonant mode, $\left|E\right|$ is the $z$ component of the THz *E*-field. **d** Dependence of the initial peak amplitude $\left|E\right|$ of the THz waves on the pump laser power, the dotted line is the linear fitting for the measured data. **e** Resonant frequency varies with the initial peak amplitude of THz waves, the dotted line is the linear fitting for the measured data

In order to explore the frequency shift caused by the TKE, experiments were performed with varying pump laser powers but with the same configuration. As is shown in Fig. 3a, time traces of the THz *E*-field at point *A* (see Fig. 2a) in the microcavity with varied pump powers are obtained. The peak amplitude of the THz *E*-field decreases as the pump power decreases, and the *E*-field decays with time. Then the spectral information in the microcavity is obtained by applying Fourier transformation to time-domain signals. The results exhibited an obvious frequency shift with the change in power of the pump pulses, as shown in Fig. 3b (top panel), indicating a positive Kerr coefficient, and the cavity Q is calculated to be about 70 for varied pump powers. Figure 3c illustrates the calculated field intensity distribution of the resonant mode in the microcavity, which is mostly localized in the cavity and remains stable as pump power increases.

Since the THz waves are generated via optical rectification^39^, the initial peak amplitude of generated THz waves is proportional to the power of the input pump pulses^40^ (see “Materials and methods”). This result is also experimentally verified, shown in Fig. 3d. The intensity of THz waves is given by:

3 $$I=\frac{1}{2}{n}_{0}{\epsilon }_{0}c{\left|E\right|}^{2}$$

where $E$ represents the amplitude of THz waves, and ${\epsilon }_{0}$ = $8.85\times {10}^{-12}\,{\rm{F}}\cdot {{\rm{m}}}^{-1}$ stands for the permittivity of vacuum. Nonlinear refractive index reads:

4 $${n}_{2}=\frac{3{\chi }^{\left(3\right)}}{2{n}_{0}^{2}{\epsilon }_{0}c}$$

here, ${\chi }^{\left(3\right)}$ is the third-order nonlinear susceptibility, so the TKE caused refractive index change is given by:

5 $$\varDelta n=\frac{3{\chi }^{\left(3\right)}}{4{n}_{0}}{\left|E\right|}^{2}$$

and resonant frequency $\nu$ is modulated by:

6 $$\frac{c}{\nu }=\frac{2}{M}\left({n}_{0}+\frac{3{\chi }^{\left(3\right)}}{4{n}_{0}}{\left|E\right|}^{2}\right)L$$

Figure 3e shows the resonant frequency varies with the terahertz electric field intensity, and the dotted line is the linear fitting for the measured data (blue point). From the linear fitting, the third-order nonlinear susceptibility can be calculated. Since THz electric field keeps decaying after generated^41^, and the spectrum is obtained by applying Fourier transformation to the whole time-domain signal of THz waves from generation to dissipation, it is logical to take the time-averaged *E*-field intensity into the calculation for the nonlinear susceptibility. In order to calculate the lower limit, the initial peak amplitudes ($\left|E\right|$) in Fig. 3d are taken into the calculation, which means the non-resonant frequencies also contribute to the frequency shift in the experiment. In fact, the non-resonant components contribute to the frequency shift during the first several round-drips after THz waves are generated. According to waveguide analysis^37^, effective linear refractive ${n}_{0}$ is 4.20 at 0.63 THz. Accordingly, the nonlinear susceptibility is calculated to be larger than $2.21\times {10}^{-15}\,{{\rm{m}}}^{2}\cdot {{\rm{V}}}^{-2}$ at 0.63 THz, which represents a value over 4 orders of magnitude larger than that for visible light in LN^42^. Notably, in our experiment, THz waves are optimized to be *z* polarized, thus this result means $\mathrm{Re}\left({\chi }_{{zzzz}}^{\left(3\right)}\right) \,>\, 2.21\times {10}^{-15}\,{{\rm{m}}}^{2}\cdot {{\rm{V}}}^{-2}$, which also shows a nonlinear refractive index of ${{n}_{2} \,>\, 7.09\times 10}^{-14}$ ${{\rm{m}}}^{2}\cdot {{\rm{W}}}^{-1}$.

To further validate the aforementioned calculations, we use the finite element method to simulate the frequency shift via COMSOL Multiphysics. In the simulation, an identical Fabry-Pérot microcavity is constructed. For the cavity, the relative permittivity is set to vary with the peak terahertz field intensity using the previously calculated nonlinear susceptibility (see “Materials and methods”), and simulations at different THz wave intensities are conducted, as shown in the bottom panel of Fig. 3b, observing that the resonant frequency experienced a redshift with the increase of THz electric field, which is in agreement with the experimental results.

The third-order nonlinearity of some substances at different frequencies is summarized in Table 1. At optical frequencies, the nonlinear refractive index is usually weak^43–46^. Recent years, it has been found that the refractive index could be perturbed by terahertz pulses^29–31,44^. However, the values for the nonlinear refractive index are generally on the same order of magnitude as values reported for all-optical measurements. For THz waves, liquids show giant Kerr coefficients^47^, owing to the vibrations of molecules. However, it is quite difficult to make practical devices with liquids. Lactose^28^ (powder) has been measured with a large nonlinear refractive index, but it is also not conducive to making practical devices. The Kerr nonlinearity can be enhanced in silicon at high THz intensities due to thermal effects or acceleration of carriers^28^, but it is still feeble and impedes practical THz applications. Quartz has a giant Kerr coefficient^48^, and may be promising for future THz applications. The Kerr coefficient of ZnSe is not particularly large^49^, but ZnSe is still a promising material for THz photonics. In this work, LN shows a giant Kerr nonlinearity attributed to SPhPs, as it has been a competitive candidate for THz generation and detection, thus indicating broad applications in the future.

**Table 1:** Nonlinearity of different substances

Frequency		Substances	Nonlinear susceptibility	
			${\boldsymbol{n}}_{\boldsymbol{2}}$ (c${{\mathbf{m}}}^{\boldsymbol{2}}\cdot {{\mathbf{W}}}^{\boldsymbol{-1}}$)	${\boldsymbol{\chi }}^{({\boldsymbol{3}})}\,({\mathbf{m}}^{\boldsymbol{2}}\cdot {\mathbf{V}}^{\boldsymbol{-2}})$
Visible/IR frequencies	800 nm	air (*g*)	$2.4\times {10}^{-19}$	$8.5\times {10}^{-26}$
		MgO (*s*)	$7\times {10}^{-17}$	$7.42\times {10}^{-23}$
		Al_2_O_3_ (*s*)	$2.93\times {10}^{-16}$	$3.21\times {10}^{-22}$
	1030 nm	fused silica (*s*)	$2.19\times {10}^{-16}$	$1.63\times {10}^{-22}$
		CaF_2_ (*s*)	$1.71\times {10}^{-16}$	$1.24\times {10}^{-22}$
	530 nm	LN (*s*)	$5.3\times {10}^{-15}$	$1.02\times {10}^{-20}$
Optical frequency & THz (pump: THz & probe: 800 nm) ${{{\chi}}^{(3)}}\left({\omega }_{{\rm{THz}}},{\omega }_{{\rm{THz}}},{\omega }_{{\rm{opt}}}\right)$		air (*g*)	$1.3\times {10}^{-19}$	$4.6\times {10}^{-26}$
		CS_2_ (*l*)	$7\times {10}^{-14}$	$2.08\times {10}^{-20}$
		CCl_4_ (*l*)	$2.7\times {10}^{-15}$	$1\times {10}^{-21}$
		MgO (*s*)	$5\times {10}^{-17}$	$5.3\times {10}^{-23}$
		Al_2_O_3_ (*s*)	$7\times {10}^{-17}$	$7.68\times {10}^{-23}$
		As_2_S_3_ (*s*)	$1.75\times {10}^{-14}$	$4.84\times {10}^{-20}$
		As_2_Se_3_ (*s*)	$3.44\times {10}^{-14}$	$9.96\times {10}^{-20}$
THz		water (*l*)	$7\times {10}^{-10}$	$1.31\times {10}^{-15}$
		ethanol (*l*)	$6\times {10}^{-9}$	$5.10\times {10}^{-15}$
		$\alpha -{\rm{pinene}}$ (*l*)	$3\times {10}^{-9}$	$2.39\times {10}^{-15}$
		lactose (*s*)	$-1.49\times {10}^{-12}$	$-1.81\times {10}^{-18}$
		silicon (*s*)	$3.51\times {10}^{-12}$	$1.44\times {10}^{-17}$
		quartz (*s*)	$7.5\times {10}^{-10}$	$1.14\times {10}^{-15}$
		ZnSe (*s*)	$4\times {10}^{-11}$	–
		LN (*s*, this work)	$> 7.09\times {10}^{-10}$ @ 0.63 THz	$> 2.21\times {10}^{-15}$ @ 0.63 THz

*s* solid, *l* liquid, *g* gas, *Optical frequency & THz* the refractive index at optical frequencies is perturbed by THz pulses, and the nonlinear susceptibility is ${\chi }^{\left(3\right)}({\omega }_{{\rm{THz}}},{\omega }_{{\rm{THz}}},{\omega }_{{\rm{opt}}})$

Theoretically, nonlinear Huang equations are employed to explain the contribution of SPhPs to such a giant TKE^33,35^:

7 $$\begin{array}{l}\ddot{x}+\gamma \dot{x}+{\omega }_{0}^{2}x={\left({Nm}\right)}^{-{1}/{2}}{bE}-\beta {x}^{3}+\left(\frac{q}{m}\right){E}_{{ex}}\\ P={\left({Nm}\right)}^{1/2}{bx}+{\epsilon }_{0}\left({\varepsilon }_{{\infty }}-1\right)E\end{array}$$

The first equation depicts how ions are driven to oscillate anharmonically when THz waves oscillate in the microcavity, where $x$, $q$, ${\omega }_{0}$ and $m$ represent the motion amplitude, electric charge, eigen angular frequency and effective mass of ions, respectively. ${E}_{{ex}}$ stands for the corresponding driving THz field, and the strength of the damping force and the anharmonicity (i.e., the nonlinearity) are characterized by parameters $\gamma$ and $\beta$, respectively. The term ${\left({Nm}\right)}^{-1/2}{bE}$ represents the contribution of SPhPs, which distinguishes the equation from classic model of the anharmonic oscillator. The second equation describes the total polarization, and the nonlinear polarization caused by the anharmonic oscillation of ions is the origin of the nonlinear phenomena, such as TKE. By solving this equation^50^, we could obtain the third-order nonlinear susceptibility (see Supplementary Note 2):

8 $${\chi }^{\left(3\right)}\left({\omega }_{1}{\rm{;}}{\omega }_{1},-{\omega }_{1},{\omega }_{1}\right)=\frac{N\beta {q}^{4}}{{m}^{3}{\epsilon }_{0}D\left({\omega }_{1}\right)D\left(-{\omega }_{1}\right)D\left({\omega }_{1}\right)D\left({\omega }_{1}\right)}$$

Take the values for an LN crystal^51^, the third-order nonlinear susceptibility at 0.63 THz is calculated to be ${\chi }^{\left(3\right)}=2.09\times {10}^{-14}\,{{\rm{m}}}^{2}\cdot {{\rm{V}}}^{-2}$, which agrees well with the experiment. For comparison, if the contribution of SPhPs is excluded, the mere ionic nonlinearity can be calculated by the classical anharmonic oscillator model for ions (see Supplementary Note 2), the third-order nonlinear susceptibility at 0.63 THz is finally calculated to be ${\chi }_{{\rm{ions}}}^{\left(3\right)}=4.38\times {10}^{-22}\,{{\rm{m}}}^{2}\cdot {{\rm{V}}}^{-2}$. Clearly, the SPhPs extremely enhanced the Kerr nonlinearity.

**Fig. 4:**
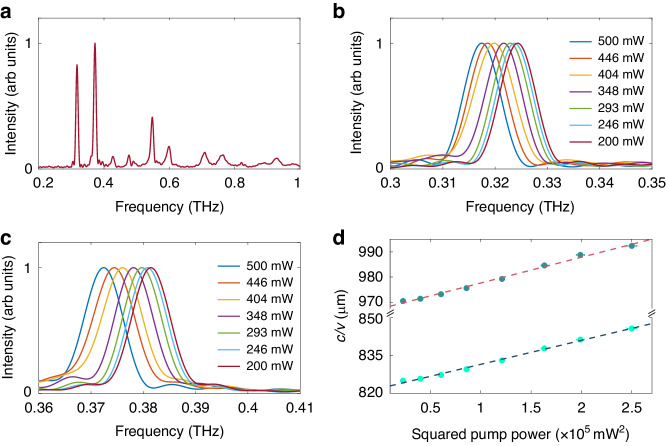
Frequency shift in a multi-mode microcavity. **a** Spectrum of the multi-mode microcavity at a low pump power. **b**, **c** Spectra of the two dominant modes in the multi-mode microcavity at different pump powers. **d** Resonant frequencies of the two dominant modes varies with the squared pump power. The dotted line represents the linear fitting for the measured data

Above all, we have demonstrated the TKE in a single-mode microcavity, while for a multi-mode microcavity, the cross-modulation between different modes also exists, except for the self-modulation. We refer to this as “hybrid modulation”, which includes both self-modulation and cross-modulation. Therefore, a microcavity with a 500 μm long cavity was fabricated, in which the width of a slot in the DBRs is 100 μm and the distance between two slots is 200 μm, and Fig. 4a shows the spectrum in it. There are two dominant modes in the microcavity, with resonant frequencies of 0.32 THz and 0.38 THz, respectively. Using the same method, we can acquire the correlation between the resonant frequencies of the two modes and the pump energy (200–500 μJ), respectively, as shown in Fig. 4b, c, and work out that the cavity Q is around 40 at 0.32 THz for varied pump powers, while around 44 at 0.38 THz.

For hybrid modulation, the modified standing wave model is still applicable:

9 $$\begin{array}{c}{M}_{1}\frac{c}{{\nu }_{1}}=2\left({n}_{0}+{n}_{11}{I}_{1}+{2n}_{12}{I}_{2}\right)L\\ {M}_{2}\frac{c}{{\nu }_{2}}=2\left({n}_{0}+{n}_{21}{I}_{1}+{2n}_{22}{I}_{2}\right)L\end{array}$$

The cross Kerr coefficients read:

10 $${n}_{{ij}}=\frac{3{\chi }^{\left(3\right)}\left({\omega }_{i}{\rm{;}}{\omega }_{j},-{\omega }_{j},{\omega }_{i}\right)}{2{n}_{0}^{2}{\epsilon }_{0}c}\,(i,\,j=1,\,2)$$

where ${\nu }_{1}$ and ${\nu }_{2}$ denote the resonant frequencies, respectively. ${I}_{1}$ and ${I}_{2}$ respectively refer to the intensity of two modes. The refractive index changes for each mode induced by the hybrid modulation are:

11 $$\varDelta {n}_{1,2}=\frac{3{\chi }^{\left(3\right)}\left({\omega }_{1,2}{\rm{;}}{\omega }_{1,2},-{\omega }_{1,2},{\omega }_{1,2}\right)}{4{n}_{0}}{\left|{E}_{1,2}\right|}^{2}+\frac{3{\chi }^{\left(3\right)}\left({\omega }_{1,2}{\rm{;}}{\omega }_{2,1},-{\omega }_{2,1},{\omega }_{1,2}\right)}{2{n}_{0}}{\left|{E}_{2,1}\right|}^{2}$$

Here, ${E}_{1}$ and ${E}_{2}$ respectively refer to the electric field amplitude of the two modes, and their peak values should also increase linearly with the pump power. The cross-modulation introduces two new tensor elements ${\chi }^{\left(3\right)}\left({\omega }_{1};{\omega }_{2},-{\omega }_{2},{\omega }_{1}\right)$ and ${\chi }^{\left(3\right)}\left({\omega }_{2};{\omega }_{1},-{\omega }_{1},{\omega }_{2}\right)$, but we can still infer that the reciprocal of the resonant frequency will increase linearly with the intensity of the THz electric field. In the experiments, since the electric field of each frequency component cannot be measured separately, only the relationship between resonant frequencies and pump power can be obtained, as depicted in Fig. 4d. The measured experimental results agree well with the expectation that the resonant frequencies are inversely proportional to the squared pump power.

## Discussion

In conclusion, we have experimentally obtained a significant THz Kerr nonlinearity due to the contribution of SPhPs. Obvious frequency shifts induced by the TKE in a single-mode microcavity under varying pump powers are observed, and the third-order nonlinear susceptibility is calculated from the frequency shifts, which is larger than $2.21\times {10}^{-15}\,{{\rm{m}}}^{2}\cdot {{\rm{V}}}^{-2}$ at 0.63 THz, exhibiting 4 orders of magnitude larger than that in visible range. The frequency shifts induced by self-modulation and cross-modulation in a multi-mode microcavity between different modes are also explored. Nonlinear Huang equations are proposed to explain the contribution of the SPhPs to the giant TKE nonlinearity, and the theoretical result agrees well with the experiments. The SPhPs enhanced TKE presents an innovative platform for a range of practical THz photonic devices, which is greatly conducive to the development of high-speed THz communication, and applicable to versatile, stable and compact THz photonic chips. In the future, the study of supercontinuum spectra could possibly expand towards the THz frequency range, which could provide the SPhPs enhanced TKE with an opportunity to exhibit their great potential in broadband terahertz waves generation. Furthermore, our work is advantageous for TKE-based physical, chemical, and biological systems.

## Materials and methods

### Generation of THz waves

In the experiment, THz waves were generated via femtosecond laser pulses induced optical rectification (OR) in LN. As a second-order nonlinear process, the nonlinear polarization can be written as^39^:

12 $${P}_{{NL}}\left(\varOmega \right)={\int }_{{\omega }_{0}-\varDelta \omega/2}^{{\omega }_{0}+{\varDelta \omega}/2}{\epsilon }_{0}{\chi }^{\left(2\right)}\left(\varOmega {\rm{;}}\varOmega +\omega,-\omega \right){E}_{p}\left(\varOmega +\omega \right){{E}_{p}}^{* }\left(\omega \right){\rm{d}}\omega$$

here, ${\chi }^{\left(2\right)}$ represents the second-order nonlinear susceptibility of LN, and ${\epsilon }_{0}$ stands for the permittivity of vacuum. ${\omega }_{0}$ and $\varDelta \omega$ are the central frequency and the spectral width of the pump laser pulse, and $\varOmega$ represents the frequency of the generated THz waves. ${E}_{p}\left(\varOmega +\omega \right)$ and ${{E}_{p}}^{* }\left(\omega \right)$ stand for the electric field of the pump laser corresponding to different frequencies. The generated THz wave is a picosecond pulse with a 0.5 THz center frequency and a frequency range of 0.2–1.2 THz.

In the experiment, the cylindrical lens we used has a focal length of 10 cm and a diameter of 2.4 cm. The theoretical limit of the excitation spot is calculated to be about 6.7 μm wide, but wider in the experiment.

### Detection of THz waves

The THz wave would cause a refractive index variation,$\,\varDelta n\left(x,z\right)$, when oscillating in the microcavity. As a result, the probe beam would gather an integral phase shift $\varDelta \phi \left(x,z\right)$ associated with the refractive index variation after propagating through the sample. The dependence of the phase shift on the THz electric field ${E}_{{THz}}\left(x,z\right)$ can be written as:

13 $$\varDelta \phi \left(x,z\right)=2\pi \frac{h}{{\lambda }_{p}}\varDelta n\left(x,z\right)=2\pi \frac{h}{{\lambda }_{p}}\frac{{n}_{e}^{3}{r}_{33}}{2}{E}_{{\rm{THz}}}\left(x,z\right)$$

where $h$ is the thickness of the sample and ${\lambda }_{p}$ represents the wavelength of the probe beam. To achieve an optimum THz signal, the pump, probe and THz waves all chose the extraordinary polarization, accordingly, ${n}_{e}$ and ${r}_{33}$ stand for the extraordinary refractive index and electro-optic coefficient of the LN crystal, respectively.

The phase-to-intensity conversion is accomplished by a phase-contrast imaging technique using a 4f system with usage of a quarter-wave phase plate (placed in the Fourier plane (focal plane) of the system). The phase-to-intensity conversion is given by:

14 $$I\left(x,z\right)={I}_{0}(x,z)\left[3-2\sqrt{2}\cos \left(\frac{\pi }{4}+\varDelta \phi (x,z)\right)\right]$$

where ${I}_{0}(x,z)$ is the intensity distribution of the origin probe beam before generation of the THz waves. It indicates that the phase shift in the sample can be obtained from the intensity distribution of the probe beam collected by the CCD camera:

15 $$\varDelta \phi \left(x,z\right)=\arccos \left[\frac{\sqrt{2}}{4}\left(3-\frac{I\left(x,z\right)}{{I}_{0}(x,z)}\right)\right]-\frac{\pi }{4}$$

According to the relation between THz electric field and the phase shift, the THz electric field distribution can be calculated:

16 $${E}_{{\rm{THz}}}\left(x,z\right)=\frac{{\lambda }_{p}}{{{hn}}_{e}^{3}{r}_{33}}\left\{\arccos \left[\frac{\sqrt{2}}{4}\left(3-\frac{I\left(x,z\right)}{{I}_{0}(x,z)}\right)\right]-\frac{\pi }{4}\right\}$$

In the experiment, for each 200-fs time delay, the CCD collected an image, so that the evolution of THz waves was recorded as a series of images. Images were saved as a data array, which are described by the reference of ${I}_{0}(x,z)$ and signals of $I\left(x,z\right)$ at different time delays. According to Eq. (16), the spatiotemporal evolution of THz waves was obtained as ${E}_{{\rm{THz}}}\left(x,z,t\right)$. When processing the data, the data of a fixed region (which is as wide as the cavity and 16 microns high) in the microcavity imaged uniformly is extracted first, and then the data is averaged along the optical axis to reduce noise.

### Numerical simulation

In the simulation, the material of LN pillars of DBRs is set to a complex refractive index of $n=5.109+0.022i$, while the refractive index of air is set to 1 and considered to have no absorption. When setting the wave equation, we choose refractive index model as the electric displacement field model for DBRs and air surrounding. A distinct wave equation is established for the cavity, and the Drude-Lorentz dispersion model is chosen for electric displacement field in the cavity. To be consistent with our previous assumption, the relative permittivity of the cavity is configured to vary with the peak terahertz field intensity. A Drude-Lorentz polarization is added in correspondence, and the resonant frequency is set as the resonant frequency of the lowest order transverse optical phonon mode in LN, that is, ${\omega }_{n}=2\,{\rm{\pi }}\times 7.6\,{\rm{THz}}$, while the damping is determined based on the experimental results obtained under the lowest pump power. A broadband THz source, emitting a Gaussian-shaped THz pulse with a center frequency of 0.527 THz and lasting of 1.2 ps, is positioned in the center of the cavity. Same as in the experiment, the electric field oscillation inside the microcavity is obtained, and the spectral information in the microcavity can be obtained by performing a Fourier transformation to the electric fields.

### Supplementary information

Supplementary Information
