# Supplementary material for: Giant Kerr nonlinearity of terahertz waves mediated by stimulated phonon polaritons in a microcavity chip
Source: Light Sci Appl. 2024 Aug 23;13:212. doi: 10.1038/s41377-024-01509-y (PMC11343743; doi:10.1038/s41377-024-01509-y)
Supplement: Supplementary file 1 — Supplementary Information [file 41377_2024_1509_MOESM1_ESM.docx]

**Supplementary Information for**

**Giant Kerr nonlinearity of terahertz waves mediated by stimulated phonon polaritons in a microcavity chip**

Yibo Huang^1,2^, Yao Lu^1,2,*^, Wei Li^3^, Xitan Xu^1,2^, Xinda Jiang^1,2^, Ruobin Ma^1,2^, Lu Chen^1^, Ningjuan Ruan^3^, Qiang Wu^1,2,*^ and Jingjun Xu^1,*^

^1^The Key Laboratory of Weak-Light Nonlinear Photonics, Ministry of Education, TEDA Applied Physics Institute and School of Physics, Nankai University, Tianjin 300457, China

^2^Shenzhen Research Institute of Nankai University, Shenzhen, Guangdong 518083, China

Full list of author information is available at the end of the article

^3^Beijing Institute of Space Mechanics & Electricity, China Academy of Space Technology, Beijing, 100094, China

**Supplementary Note 1: Sample fabrication**

The lithium niobite (LN) Fabry-Perot microcavity utilized in our experiment is fabricated using a chemically assisted femtosecond-laser direct writing technique [1]. Figure S1 depicts the schematic diagram of the experimental laser-machining setup for the sample fabrication. The machining laser (800 nm central wavelength, 500 μJ per pulse, 1 kHz repetition rate, 120 fs pulse duration) first passes through a shutter, which is used to block the laser when moving from one machining region to the next, and then passes through a power control system composed of a half-wave plate (HWP) and a Glan-Taylor prism (GTP). After passing through a dichroic mirror (DM), the laser is focused on a *x*-cut LN slab coated with 1 μm thick silicon dioxide on both surfaces through an objective, wherein the sample is mounted on an *xyz*-translation stage with computer-controlled actuators. The air slots are cut out by focusing the laser at multiple depths spanning from the front to the back. To monitor the machining in real time, a beam of incoherent light illuminates the sample and is imaged on a CCD camera.

After the machining, the sample is immersed in hydrofluoric acid solution for 20-30 minutes to remove the coating along with accumulated debris. A microscope image of a microcavity we used in the experiment is shown in Fig.S2. The width of an air slot is set to be 50 μm, the width of a LN pillar between two slots is also set to be 50 μm, and the length of the cavity is set to be 245 μm. The machining error is approximately 2 microns, which has negligible effects on the experiment.


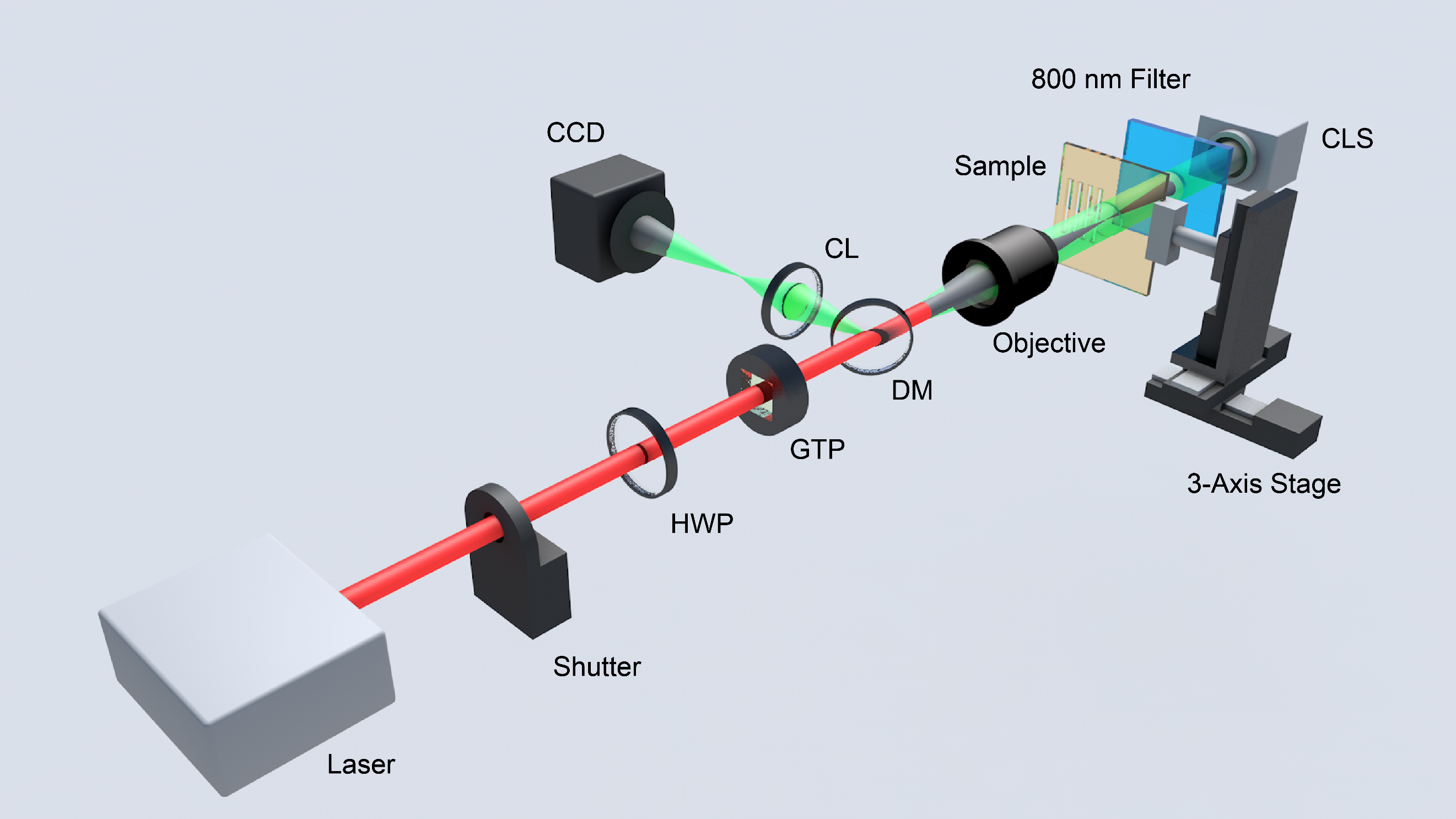


**Fig. S1. Schematic diagram of the experimental laser-machining set-up for the sample fabrication.** HWP: half-wave plate, GTP: Glan-Taylor prism, DM: dichroic mirror, CL: convex lens, CLS: cold light source.


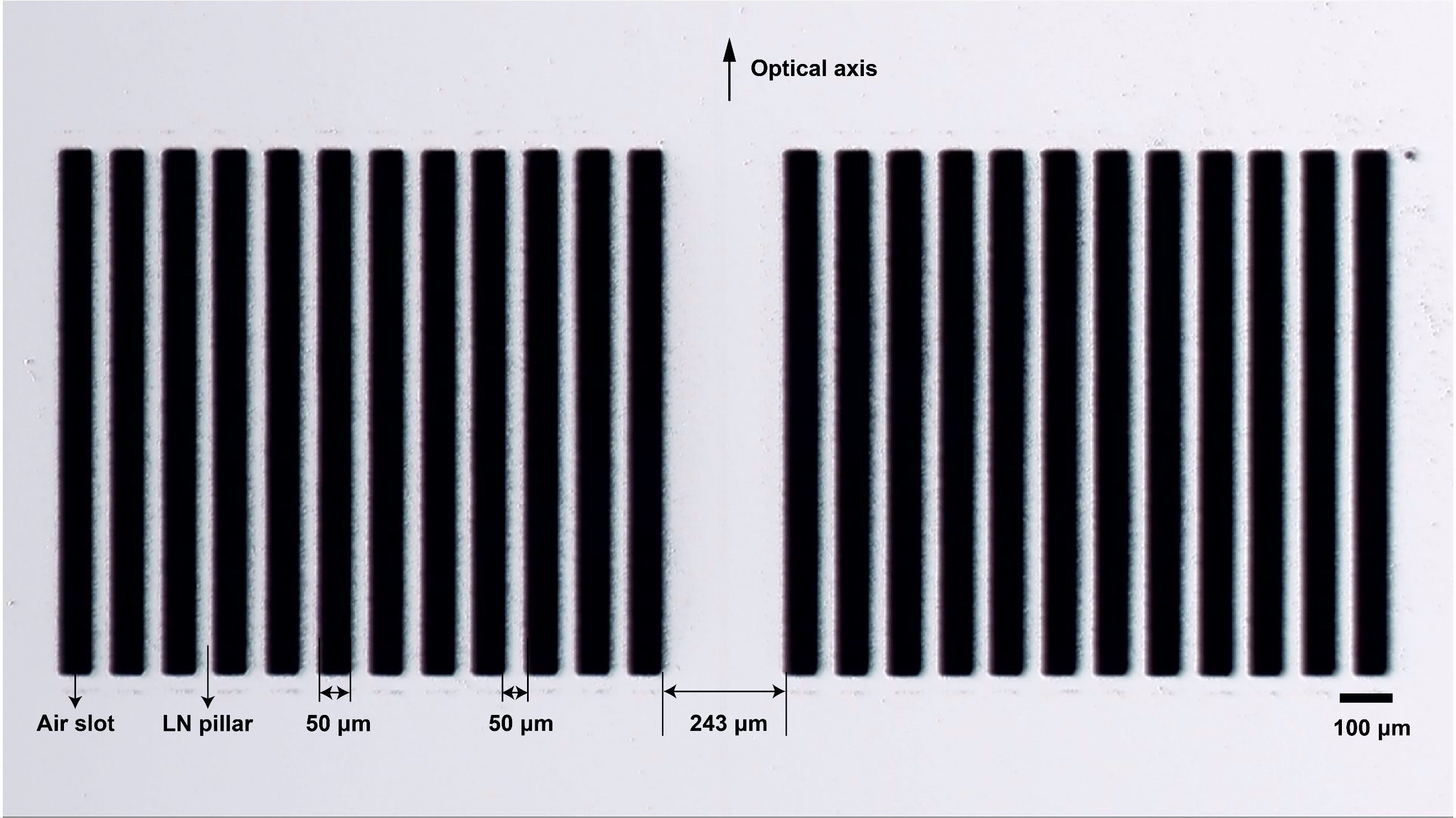


**Fig. S2. Microscope image of the microcavity used in the experiment.** The air slot and LN pillar are designed to have a width of 50 microns, and the cavity is intended to have a length of 245 microns, while the machining error is about 2 microns.

**Supplementary Note2: Theoretical analysis of phonon-polariton enhanced optical Kerr nonlinearity with modified Huang equations.**

Under an external driving of the THz wave, and along with the contribution of phonon-polaritons, the vibration equation of ions is written as follows:

$$\begin{aligned} \begin{matrix} \ddot{x}+\gamma\dot{x}+\omega_{0}^{2}x=\left( Nm \right)^{-1/2}bE-\beta x^{3}+\left( \frac{q}{m} \right)E_{ex} \\ P=\left( Nm \right)^{1/2}bx+\epsilon_{0}\left( \varepsilon_{\infty}-1 \right)E \end{matrix}\#\left( S1 \right) \end{aligned}$$

where $x$ represents the motion amplitude of ions. The electric charge, eigen angular frequency and effective mass of ions are indicated by $q$, $\omega_{0}$ and $m$. $E_{ex}$ stands for the corresponding driving THz field, and the strength of the damping force and the anharmonicity (i.e., the nonlinearity) are characterized by parameters $\gamma$ and $\beta$, respectively. The contribution of phonon polariton is the term $\left( Nm \right)^{-1/2}b_{12}E$, and the values of the coupling coefficients are determined by $b=\omega_{0}\sqrt{\epsilon_{0}\left( \varepsilon_{0}-\varepsilon_{\infty} \right)}$. $\epsilon_{0}$ is the permittivity of vacuum, $\varepsilon_{0}$ and $\varepsilon_{\infty}$ stand for the low- and high-frequency relative permittivity of the material, while $N$ is the number of oscillators per unit volume. $E$ represents the macroscopic electric field radiated by the ions. Cooperating with Maxwell’s equations, the polarization *P* can be eliminated from the above equations:

$$\begin{aligned} \epsilon_{0}\mu_{0}\varepsilon_{\infty}\ddot{E}+\mu_{0}\left( Nm \right)^{1/2}b\ddot{x}-\nabla^{2}E=0\#\left( S2 \right) \end{aligned}$$

The slow time approximation is adopted, that is, assuming $x=x_{0}ⅇ^{-j\omega t}$ and $E=E_{0}ⅇ^{-j\omega t}$ with $\omega$ being the angular frequency of the driving THz field, where $x_{0}$ and $E_{0}$ are independent of $t$.

We assume that the propagation of $E$ in space is described by $ⅇ^{j\sqrt{\varepsilon_{0}}r}$ (actually should be $e^{j\sqrt{\varepsilon_{r}}kr}$, but for waves of 0.2-1.2 THz, $\varepsilon_{r}\sim\varepsilon_{0}$, the error from this assumption is negligible), then we take $\nabla^{2}E=-{\varepsilon_{0}k}^{2}E=\omega^{2}/c^{2}E$ into S2. Here, $c=\frac{1}{\sqrt{\epsilon_{0}\mu_{0}}}$ indicates the light speed in the vacuum and $\mu_{0}$ is the permeability of the vacuum. The following equation can be obtained:

$$\begin{aligned} \left( Nm \right)^{-1/2}bE=\omega_{0}^{2}x\#\left( S3 \right) \end{aligned}$$

We need to point out: since it is an approximation based on the assumption above, we consider that this approximation is valid under the driving of relatively strong external THz waves. Under the driving of external THz waves, the difference between the coupling term and restoring force term, $\left( Nm \right)^{-1/2}bE-\omega_{0}^{2}x$, can be approximated as 0. If there are no external THz waves or the THz waves are weak, or the frequency is so high that the assumption is invalid, this approximation is no longer applicable, which means that $\left( Nm \right)^{-1/2}bE-\omega_{0}^{2}x$ cannot be ignored.

Finally, taking S3 into S1, a Drude dispersion equation is obtained:

$$\begin{aligned} \ddot{x}+\gamma\dot{x}=-\beta x^{3}+\left( \frac{q}{m} \right)E_{ex}\#\left( S4 \right) \end{aligned}$$

This equation indicates an impressive delocalization of phonon-polaritons, and the mobility of phonon-polaritons is comparable to that of the free electrons in a metal. The ionic nonlinearity can be prodigiously enhanced because of phonon-polaritons.

Using the conventional method in nonlinear optics [2], we seek a solution having the form of a power series in parameter $\xi$:

$$\begin{aligned} x=x^{\left( 1 \right)}+\xi x^{\left( 2 \right)}+\xi^{2}x^{\left( 3 \right)}+\cdots\#\left( S5 \right) \end{aligned}$$

We insert this equation into the Drude dispersion equation, while retaining the terms up to $\xi^{2}$, then get three equations:

$$\begin{aligned} \ddot{x}^{\left( 1 \right)}+\gamma\dot{x}^{\left( 1 \right)}=\frac{qE_{ex}}{m}\#\left( S6 \right) \end{aligned}$$

$$\begin{aligned} \ddot{x}^{\left( 2 \right)}+\gamma\dot{x}^{\left( 2 \right)}=0\#\left( S7 \right) \end{aligned}$$

$$\begin{aligned} \ddot{x}^{\left( 3 \right)}+\gamma\dot{x}^{\left( 3 \right)}+\beta\left( x^{\left( 1 \right)} \right)^{3}=0\#\left( S8 \right) \end{aligned}$$

We can get the solutions as follows:

$$\begin{aligned} x^{\left( 1 \right)}\left( \omega_{1},t \right)=\frac{qE_{0}\left( \omega_{1} \right)}{mD\left( \omega_{1} \right)}\#\left( S9 \right) \end{aligned}$$

$$\begin{aligned} x^{\left( 2 \right)}\left( \omega_{1},t \right)=0\#\left( S10 \right) \end{aligned}$$

$$\begin{aligned} x^{\left( 3 \right)}\left( \omega_{4},t \right)=\sum_{1,2,3} \frac{\beta q^{3}E_{0}\left( \omega_{1} \right)E_{0}\left( \omega_{2} \right)E_{0}\left( \omega_{3} \right)}{m^{3}D\left( \omega_{4} \right)D\left( \omega_{1} \right)D\left( \omega_{2} \right)D\left( \omega_{3} \right)}e^{-ⅈ\omega_{4}t}\#\left( S11 \right) \end{aligned}$$

Here,$D\left( \omega_{1} \right)$=$-\omega_{1}^{2}-i\gamma\omega_{1}$, $\omega_{1}$,$\omega_{2}$, $\omega_{3}$ and $\omega_{4}$ are the angular frequency of the driving THz field, and $\omega_{4}=\omega_{1}+\omega_{2}+\omega_{3}$. The amplitude of the polarization component oscillation at frequency $\omega_{4}$ is given in terms of this amplitude by

$$\begin{aligned} P^{\left( 3 \right)}=-Nqx^{\left( 3 \right)}\left( \omega_{4},t \right)\#\left( S12 \right) \end{aligned}$$

And the definition of the third-order nonlinear susceptibility is

$$\begin{aligned} P^{\left( 3 \right)}=\sum_{1,2,3} \epsilon_{0}\chi^{\left( 3 \right)}\left( \omega_{4};\omega_{1},\omega_{2},\omega_{3} \right)E\left( \omega_{1} \right)E\left( \omega_{2} \right)E\left( \omega_{2} \right)\#\left( S13 \right) \end{aligned}$$

Based on the equations above, we finally get the third-order nonlinear susceptibility

$$\begin{aligned} \chi^{\left( 3 \right)}\left( \omega_{4};\omega_{1},\omega_{2},\omega_{3} \right)=\frac{N\beta q^{4}}{m^{3}\epsilon_{0}D\left( \omega_{4} \right)D\left( \omega_{1} \right)D\left( \omega_{2} \right)D\left( \omega_{3} \right)}\#\left( S14 \right) \end{aligned}$$

We assume that the harmonic and anharmonic term in the potential energy should be comparable in magnitude, so that we can get the parameter

$$\begin{aligned} \beta=\omega_{0}^{2}N^{2/3}\#\left( S15 \right) \end{aligned}$$

Take the values for an LN crystal, $\omega_{0}=2\pi\times7.6 \mathrm{THz}$, $N=6.29\times{10}^{27} m^{-3}$ and $\gamma=2\pi\times0.84 \mathrm{THz}$, the reduced mass $m=35 u$, $1 u=1.66\times{10}^{-27} \mathrm{kg}$, and the charge *q*= 5 $e$, where $ⅇ=1.6\times{10}^{-19} C$ is the elementary charge. The third-order nonlinear susceptibility at 0.63 THz is calculated to be $\mathrm{Re} \left( \chi^{\left( 3 \right)} \right)=2.09\times{10}^{-14} m^{2}\cdot V^{-2}$.

For comparison, if neglecting the contribution of phonon-polaritons, the lattice vibration is described by the classical anharmonic oscillator model:

$$\begin{aligned} \ddot{x}+\gamma\dot{x}+\omega_{0}^{2}x+\beta x^{3}=\left( \frac{q}{m} \right)E_{ex}\#\left( S16 \right) \end{aligned}$$

Using the same method as above, we finally get the third-order nonlinear susceptibility

$$\begin{aligned} \chi^{\left( 3 \right)}\left( \omega_{4};\omega_{1},\omega_{2},\omega_{3} \right)=\frac{N\beta q^{4}}{m^{3}\epsilon_{0}D\left( \omega_{4} \right)D\left( \omega_{1} \right)D\left( \omega_{2} \right)D\left( \omega_{3} \right)}\#\left( S17 \right) \end{aligned}$$

But here, $D\left( \omega_{1} \right)=\omega_{0}^{2}-\omega_{1}^{2}-i\gamma\omega_{1}$, and the parameters are all the same as above. The third-order nonlinear susceptibility without the contribution of phonon polaritons at 0.63 THz is calculated to be $Re \left( \chi^{\left( 3 \right)} \right)=4.38\times{10}^{-22} m^{2}\cdot V^{-2}$. Our results align more with the enhanced susceptibility of phonon-polaritons rather than mere ionic nonlinearity, suggesting that the giant nonlinearity originates from the strong coupling between phonons and light.

**Supplementary Note 3: Evolution of THz waves**

Using the pump-probe phase-contrast imaging system, the evolution of THz waves in the sample can be recorded (in the form of images captured by the CCD camera). Figure S3 shows the evolution of THz waves in the single-mode microcavity during the first several picoseconds after generation. As shown in Fig. S3a, the THz wave is generated in the middle of the cavity, and it propagates to both sides immediately after generation. After about 1 ps, it reaches the edges of the cavity and reflected at the edges, as shown in Fig. S3b & c. In the process of the first reflection, non-resonant part of the THz wave (not all) escapes from the cavity and propagates in the DBRs. After the first reflection, the THz wave propagates and is reflected again, showing in Fig. S3d & e. After the first several round-drips, most of the non-resonant part escapes from the cavity, and thus the cavity mode is produced. According to the Method section, the *E*-field of THz waves could be extracted from the images, i.e. the time-domain signal. When processing the data, the data of a fixed region in the microcavity that was imaged uniformly (the area surrounded by the red rectangular box in Fig. S3, which is as wide as the cavity and 16 microns high) was extracted first, and then the data was averaged along the optical axis to reduce noise.


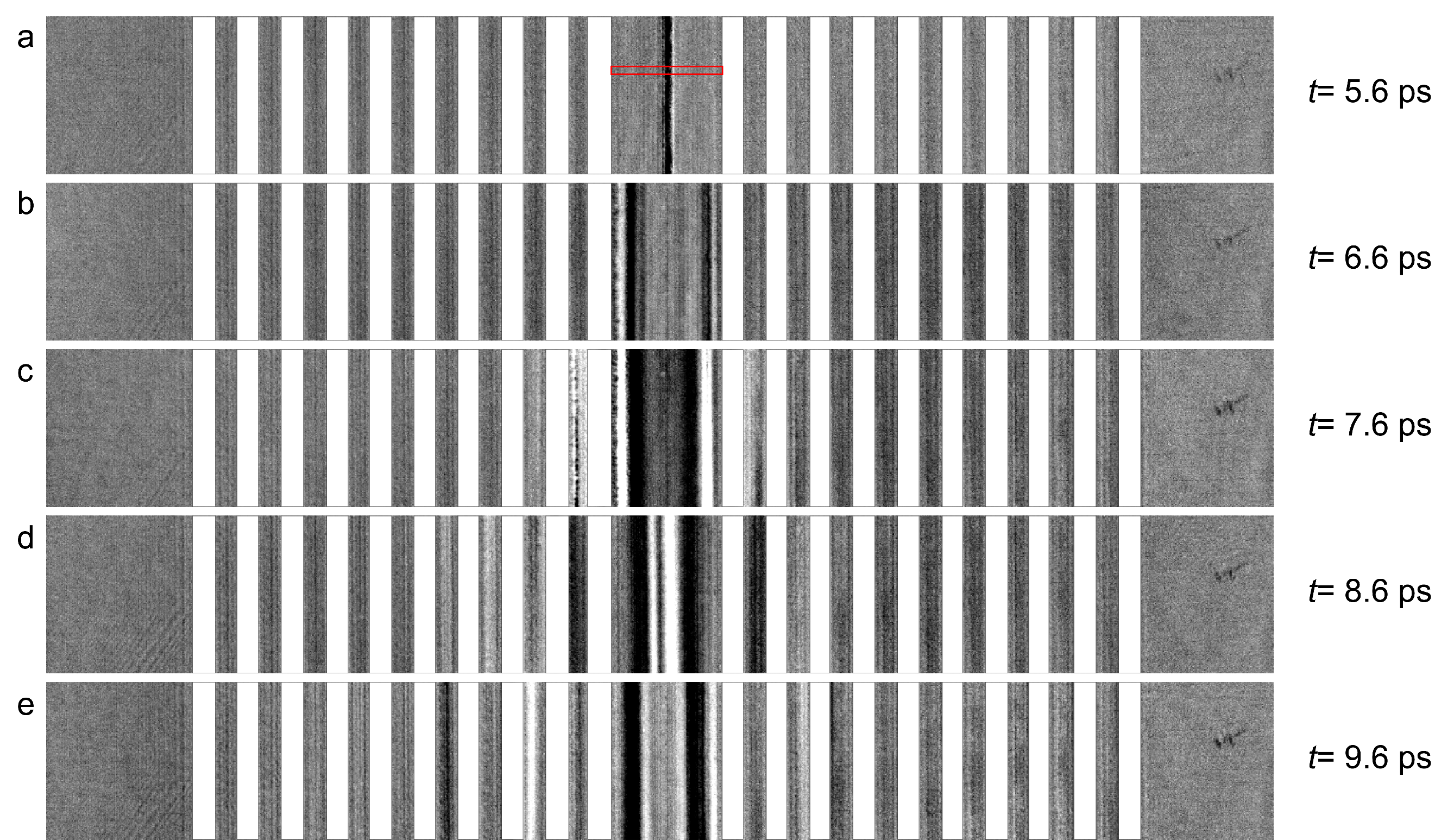


Fig. S3. Evolution of THz waves in the single-mode microcavity during the first several picoseconds after generation. Red rectangular box: region to extract data.

We also simulated the evolution of the THz field in the single-mode microcavity during the first tens of picoseconds after generation, as shown in Fig. S4. The simulation is consistent with the experiment in that the non-resonant component exits the cavity on reflection, leaving the resonant component, and the cavity mode profile gradually forms.


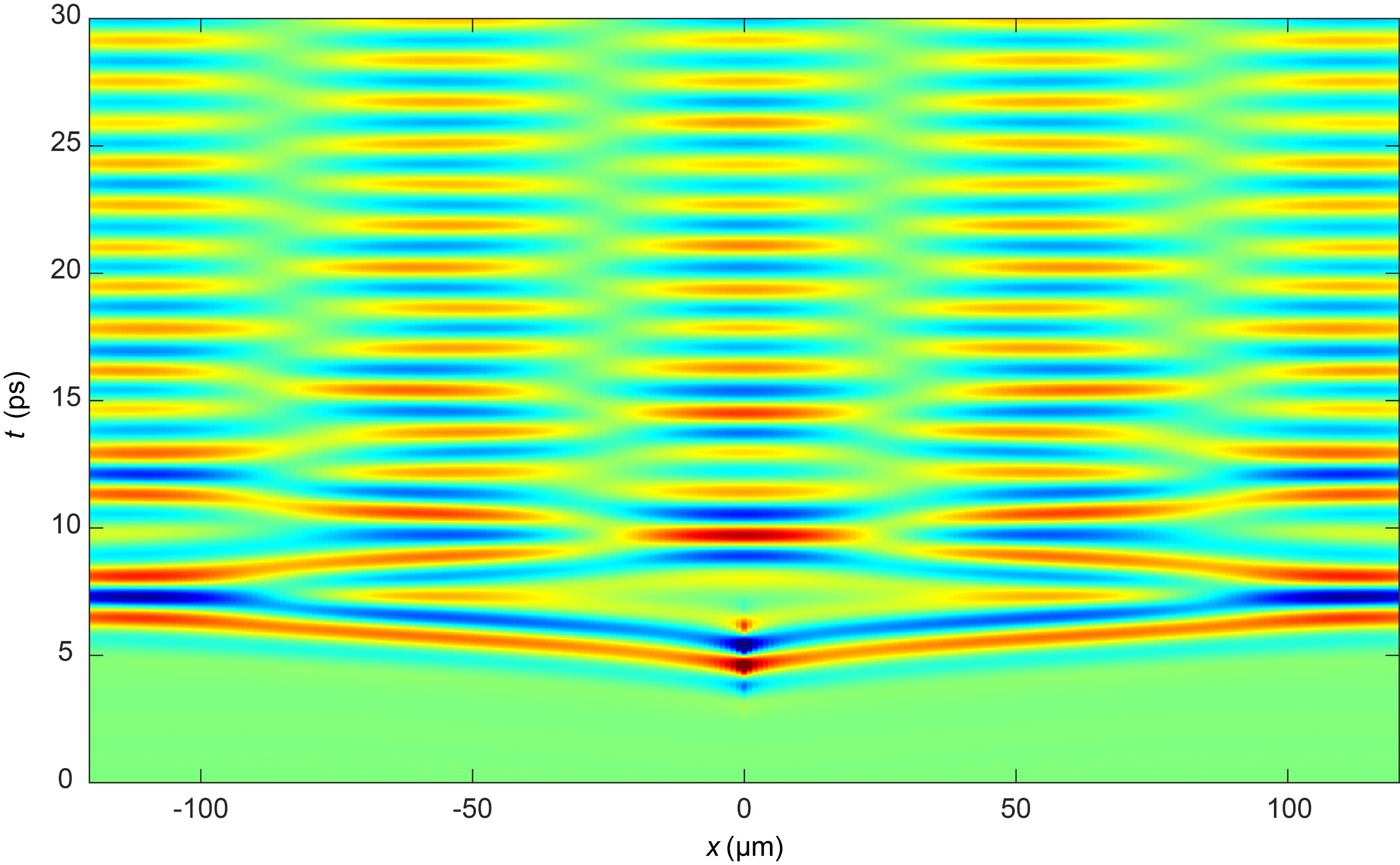


Fig. S4. Evolution of THz waves in the single-mode microcavity during the first tens of picoseconds after generation.

**References**

1. Sivarajah, P. et al. Chemically assisted femtosecond laser machining for applications in LiNbO_3_ and LiTaO_3_. *Applied Physics A-Materials Science & Processing* **112**, 615-622 (2013).

2. Boyd, R. W. Nonlinear Optics. 2nd edn. (Amsterdam: Elsevier, 2003).
